# Upregulation of MicroRNA-214 Contributes to the Development of Vascular Remodeling in Hypoxia-induced Pulmonary Hypertension Via Targeting CCNL2

**DOI:** 10.1038/srep24661

**Published:** 2016-07-06

**Authors:** HaiTao Liu, Yin Tao, Mai Chen, Jin Yu, Wei-Jie Li, Ling Tao, Yan Li, Fei Li

**Affiliations:** 1 Department of Cardiology, Xijing Hospital, Fourth Military Medical University, Xi’an, China

## Abstract

Hypoxia-induced pulmonary hypertension (PH), which is characterized by vascular remodeling of blood vessels, is a significant complication of chronic obstructive pulmonary disease (COPD). In this study, we screened 13 candidate miRNAs in pulmonary artery smooth muscle cells (PASMCs) harvested from COPD patients with PH (n = 18) and normal controls (n = 15) and found that the expression of miR-214 was differentially expressed between these two groups. Additionally, cyclin L2 (CCNL2) was validated as a target of miR-214 in PASMCs using a luciferase assay. Based on real-time PCR, immunohistochemistry and western blot, the expression of CCNL2 was substantially downregulated in PASMCs from COPD patients with PH compared with those from normal controls. Moreover, the relationship between miRNA and mRNA expression was confirmed using real-time PCR and western blot in PASMCs transfected with miR-214 mimics. Furthermore, the introduction of miR-214 significantly promoted the proliferation of PASMCs by suppressing cell apoptosis, and this effect was mediated by the downregulation of CCNL2. Exposure of PASMCs to hypoxia significantly increased the expression of miR-214, decreased the expression of CCNL2, and promoted cell proliferation. However, these effects were significantly attenuated by the introduction of miR-214 inhibitors, which significantly downregulated miR-214 expression and upregulated CCNL2 expression.

Pulmonary hypertension (PH) is a serious and occasionally fatal medical condition that is characterized by vasoconstriction and vascular remodeling, resulting in increased vascular resistance and right ventricular dysfunction^1^. Histologically, PH is a panvasculopathy that involves various vascular cell types, such as endothelial cells, smooth muscle cells and fibroblasts, and this vascular pathology can be triggered by a wide spectrum of genetic and environmental stimuli, including hypoxia^2^. As the major component of the vasculature, pulmonary artery smooth muscle cells (PASMCs) play an essential role in the response to hypoxia, and dysregulation of their activity is closely related to the development of PH. Recently, it has been shown that the enhanced proliferation of PASMCs, triggered by chronic exposure to hypoxia, is a major contributor to the development of hypoxic PH^3^.

MicroRNAs (miRNAs) are small, noncoding RNAs (21–23 nucleotides in length) that mediate post-transcriptional gene silencing^4^. Following transcription and processing in the nucleus, mature miRNAs downregulate the expression of specific target messenger RNAs (mRNAs) via Watson-Crick nucleotide binding to a “seed sequence”, which is generally located in the 3′ untranslated region (3′UTR) of mRNA, leading to a reduction in the target gene transcript level through either translational repression or transcript degradation^5^. It has been estimated that approximately 1,400 distinct miRNAs are predicted to be encoded by the human genome^6^, and those miRNAs can directly regulate at least 30% of the genes in the human genome^7^. Therefore, miRNAs are believed to be involved in the control of nearly all physiological and pathological cellular processes.

PH has been diagnosed in more than 30% of patients with COPD^8^, and those patients tend to have more severe airway obstruction, more hypoxia, less hypercapnia, and significantly compromised survival^9^. Generally regarded as a separate entity, arterial remodeling, which is the structural basis of PH, is thought to result from hypoxia caused by associated lung diseases, such as COPD. In turn, PH worsens the lung disease, creating a vicious, life-threatening cycle^10^. Recent studies of miRNAs demonstrated that these molecules may play substantial and important roles in the molecular mechanisms underlying the physiological and pathophysiological adaptations to hypoxia. Among miRNAs, those whose expression is dynamically altered by hypoxia are called “hypoxamiRs”^11^, and upregulation or downregulation of hypoxamiRs has been implicated in the development of hypoxia-induced PH, a major complication of COPD, by suppressing target gene expression or releasing the physiologic inhibition of the expression of certain genes^12^.

To explore the role of candidate miRNAs in hypoxia-induced PH, we performed quantitative real-time PCR-based screening for differentially expressed miRNAs and identified miR-214 as the only significantly upregulated miRNA in PASMCs harvested from COPD patients with PH. The objectives of the current study were to determine whether differentially expressed miR-214 is responsible for the abnormally enhanced proliferation of PASMCs and to identify the molecular mechanism underlying the aberrant enhancement of PASMC proliferation.

## Materials and Methods

### Patient samples

The study population comprised 18 patients with COPD and PH, 15 with COPD without PH, and 3 with non-familial PH without COPD, all of whom underwent a pneumonectomy (lung resection) for the treatment of a lung tumor in our hospital. Lung tissue samples were obtained from normal-appearing areas of the pulmonary parenchyma in a region as far as possible from the tumor (at least 2 cm) that was free of pleura or large airways. The clinicopathological characteristics of each patient in each group are provided in Table 1. All patients were regarded as clinically stable because none had required medical attention or exhibited any change in their standard therapy within the 3 months prior to enrollment. This study was conducted in compliance with the Declaration of Helsinki, the study protocols were approved by the research ethics committee of the Fourth Military Medical University, and all participants provided written, informed consent to participate in this study.

### Cell culture

Pulmonary artery segments that were 50–500 μm in external diameter were collected from each participant and incubated in Hanks’ solution containing 1.5 mg/mL collagenase for 20 minutes. A thin layer of adventitia was removed with fine forceps, and the intimal surface was then gently scratched with a surgical blade to remove the endothelium. Both 2.0 mg/ml collagenase and 0.5 mg/ml elastase were added to the remaining smooth muscle for digestion at 37 °C for 35–45 minutes. The cells were incubated in Dulbecco’s modified Eagle’s medium (DMEM) (Life Technologies Inc., USA) containing 10% fetal bovine serum, 100 U/mL penicillin, and 100 mg/mL streptomycin at 37 °C in a humidified incubator. After incubation, the cells were passaged via trypsinization with 0.05% trypsin–EDTA and used in experiments.

### Flow cytometry analysis

The DNA contents of the cells were determined by flow cytometry to estimate the percentages of cells in different phases of the cell cycle. The treated cells were collected, washed twice with cold PBS, and fixed in 70% ethanol at −20 °C overnight. The cells were resuspended in PBS containing 50 mg/ml propidium iodide (PI) and 10 mg/ml DNase-free RNase immediately before flow cytometry. A FACSCalibur system (Becton Dickinson, San Diego, CA, USA) equipped with CellQuest software was used for flow cytometry analysis.

For apoptosis analysis, PASMCs were seeded at 3** × **10^3^ cells/well in 96-well plates using triplicate wells for each transfection. After 48 h, the cells treated under various conditions were incubated in 1.5% H_2_O_2_ for 24 h. The cells were then starved in medium containing 0.1% FBS; stained with Annexin V-FITC and PI reagents in binding buffer using the Annexin V-FITC/PI cell apoptosis detection kit (Beyotime Institute of Biotechnology, Beijing, China); and analyzed using a FACSCalibur flow cytometer.

### Luciferase assay

**Figure 1:**
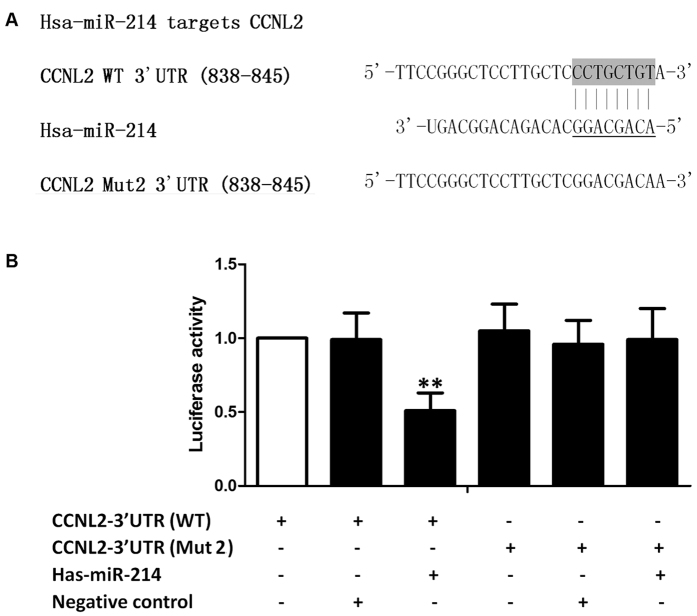
(**A**) Schematic description of mature has-miR-214 and wild-type/mutant CCNL2; (**B**) The luciferase activities of PASMCs cotransfected with miR-214 mimics and wild-type CCNL2 were significantly lower than other groups. (**P < 0.05 compared with the scramble controls).

The 3′UTR of cyclin L2 (CCNL2) was amplified via PCR and subcloned into pcDNA3 (Invitrogen, Carlsbad, CA, USA). Mutations were introduced using a site-directed mutagenesis kit (Stratagene, La Jolla, CA), and the sequence accuracy of the inserts was confirmed using direct Sanger sequencing prior to the subsequent assays. For the luciferase reporter assay, PASMCs were seeded at a density of 1** × **10^4^ cells/well in 96-well culture plates and transiently transfected with constructs as described in Fig. 1. Briefly, 100 ng of the wild-type or mutant reporter luciferase expression plasmid and 10 ng of the Renilla luciferase expression vector pRL-TK (Promega, Madison, WI) as an internal control were transfected into the PASMCs. Firefly and Renilla luciferase activities were measured using a Dual-Luciferase Reporter Assay System (Promega, Madison, WI), and firefly luciferase activity was normalized to the activity of the Renilla luciferase control.

### Oligonucleotide transfection

The miR-214 inhibitor, the miR-214 mimic and negative controls were obtained from Shanghai GenePharma (Shanghai, China). Transfection of primary PASMCs was performed using Lipofectamine 2000 (Invitrogen, Carlsbad, CA) according to the manufacturer’s instructions. For transfection, chemosynthesized siRNA (anti-CCNL2 siRNA: 5′-CTCGGAAAAAGGTTGATCTC-3′), an miR-214 mimic (5′-ACAGCAGGCACAGACAGGCAGU-3′), an miR-214 inhibitor (5′-ACUGCCUGUCUGUGCCUGCUGU-3′), or a scramble control (5′-CAGUACUUUUGUGUAGUACAA-3′) was dissolved in RNase-free water to a concentration of 20 mM to prepare a stock solution. PASMCs were plated at a density of 1.2** × **10^5^ cells/well in 6-well culture plates, and transfection with siRNA, miRNA mimics/inhibitors or the scramble control at a final concentration of 50 nM or 100 nM was performed after 24 h in culture. The cells were allowed to grow for an additional 48 h after transfection and were then treated as indicated below.

### RNA isolation and real-time PCR

Total RNA was isolated using RNAiso (TaKaRa, Dalian, China) according to the manufacturer’s instructions. The integrity of isolated RNA was determined by electrophoresis, and the amount of RNA was quantitatively determined using a NanoDrop spectrophotometer (Thermo Scientific, NJ, USA). Real-time PCR assays were performed to determine the expression levels of miRNAs or mRNAs. Briefly, approximately 200 ng of total RNA was converted to cDNA, and the cDNA was quantified using SYBR Green Realtime PCR Master Mix (QPK-201, Toyobo, Tokyo, Japan) in an ABI 7900 PCR system (Applied Biosystems, Foster City, CA). Data analysis was performed using the 2^−ΔΔCt^ method. The results were normalized to the mRNA levels of U6.

### Western blot

Cells were lysed in lysis buffer, and the protein content was determined using a BCA kit (Pierce, Rockford, IL, USA). Five micrograms of protein per lane were separated on a 10% sodium SDS-polyacrylamide gel and transferred to a PVDF membrane (Millipore, Billerica, MA, USA). The membrane was blocked for 1 h (in 5% non-fat milk powder in TBS-T) and incubated overnight with an anti-CCNL2 (dilution 1:2000) or anti-caspase-3 primary antibody (all antibodies were purchased from Santa Cruz Biotechnologies, Santa Cruz, CA). The membranes were then washed and incubated for 2 h in a secondary antibody (goat anti-rabbit 1:10000, Santa Cruz, CA). The signals were detected using ECL Plus (Pierce, Rockford, IL, USA), and the band intensities of target proteins were quantified via densitometry using ImageQuant software.

### Exposure of PASMCs to hypoxia

PASMCs were exposed to hypoxia, which was generated in a chamber equilibrated with a water-saturated gas mixture of 1% O_2_, 5% CO_2_ and 94% N_2_ at 37 °C for 24 h, as described previously^12^.

### Caspase-3 activity assay

The caspase-3 activity assay was determined using a Caspase-Glo 3 Assay kit (Promega, Madison, WI) according to the manufacturer’s instructions.

### Cell counting

The same numbers of cells treated under various conditions were plated in 6-well plates (5000 cells/well) in SmGM-2, and the adherent cells (viable cells) were detached via treatment with trypsin and then counted using a TC10 Automated Cell Counter (Bio-Rad, Hercules, CA). All experiments were performed in triplicate.

### BrdU incorporation assays

BrdU was added 2 h before culture termination. At the end of the culture period, cells were blocked with goat serum and incubated overnight at 4 °C in an anti-BrdU antibody. Then, the cells were incubated for 60 min in biotinylated goat anti-mouse IgG (1:200) and stained with DAB. The percentage of stained cells was determined by counting the number of positively stained cells within a microscopic visual field and dividing that number by the total number of cells in the same field.

### Immunohistochemistry

The removed lung tissues were fixed with 4% paraformaldehyde. To determine the expression level of CCNL2, paraffin-embedded sections were incubated in the anti-CCNL2 antibody (Santa Cruz, CA) at a dilution of 1:200. After overnight incubation at 4 °C, the slides were washed three times with PBS and then incubated in the REAL EnVision Detection System (Dako Diagnostics, Shanghai, China).

### Plasmid vector construction

The oligonucleotide sequence of anti-miR-214 and a negative control sequence were chemically synthesized by GenePharma (Shanghai, China) and inserted into the pGPU6/GFP vector.

### Animal experiments

The hypoxic PH animal model was established as described previously^13^. Male mice (BL6) were obtained by the Laboratory Animal Center of the Fourth Military Medical University. Three animal groups were used (8 animals in each group): one group was exposed to normoxic conditions, and two groups (treated with either anti-miR-214 or the negative control) were exposed to hypoxic conditions. The oxygen fraction was decreased gradually from 21 to 10% over 60 min. Hypoxic conditions were provided by chambers connected to a gas mixer (Ruskinn Life Science). This study was conducted in compliance with the regulations of the university, and the protocols were approved by the research ethics committee of the Fourth Military Medical University.

For gene delivery *in vivo*, the anti-miR-214 plasmid or the negative control plasmid was mixed with EXGEN500 and 5% glucose (Fermentas Biotechnology Co., Ltd., Burlington, Ontario, Canada). Mice were intratracheally treated with the oligonucleotides/EXGEN500 complex (at a ratio of 100 μg/330 μl). Additionally, mouse PASMCs were isolated from the pulmonary artery, and the transfection efficiency was estimated to be 95% as demonstrated by the percentage of GFP-positive cells based on flow cytometry (data not shown).

### Statistical analysis

The independent t-test was used to compare samples with a parametric distribution between two groups. For statistical analysis between more than two groups, one-way ANOVA was used. In all statistical analyses, the data are expressed as the means ± standard deviation (SD). GraphPad Prism Software (GraphPad Software, San Diego, CA, USA) was used for statistical analysis, and a P-value < 0.05 was considered to be statistically significant (*P < 0.05, **P < 0.01).

## Results

### The expression level of miR-214 is significantly upregulated in human PASMCs (hPASMCs) harvested from COPD patients with PH compared to hPASMCs harvested from COPD patients without PH

**Figure 2:**
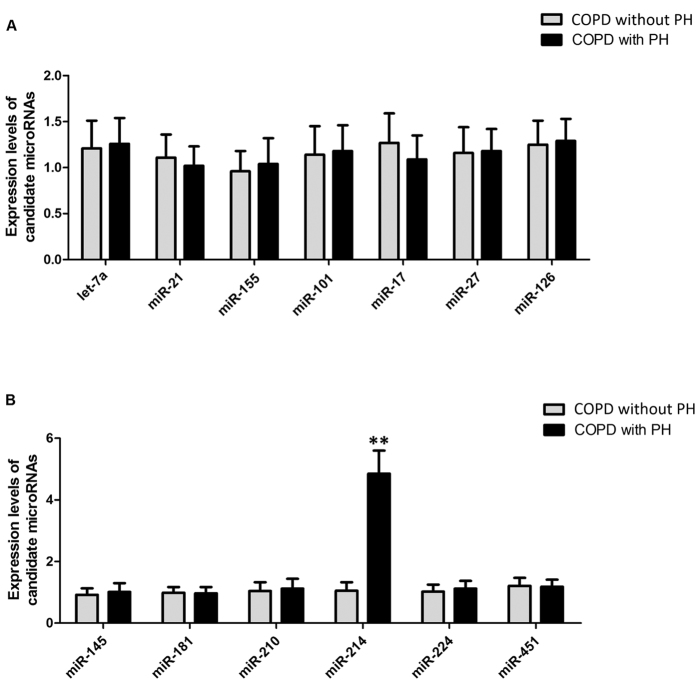
Real-time based screening to identify the differentially expression miRNA among those that have been previous reported to be functionally associated with hypoxia exposure, and we found that miR-214 was the only differentially expressed miRNA and its expression was significantly upregulated in PASMCs collected from COPD patients with PH compared with the normal controls. (**P < 0.05 compared with the scramble controls).

To identify the miRNA that is responsible for the development of hypoxia-induced PH, such as PH in patients with COPD, we quantified the expression levels of thirteen candidate miRNAs that have been reported to be associated with hypoxia in previous publications^12^,^14^ and compared the expression patterns of those miRNAs in hPASMCs from COPD patients with and without PH. As shown in Fig. 2A,B, the quantitative real-time PCR results showed that the expression levels of miR-214 were significantly higher in hPASMCs from COPD patients with PH than in hPASMCs from COPD patients without PH. The clinicopathological data for the participants are shown in Table 1. No difference in age, gender, height, weight or BMI was noted between the cases and the controls. As expected considering the recruitment criteria, FEV1, DLCO, TLC and FVC were significantly lower in the case group than in the control group.

### Direct targeting of miR-214

Using www.mirdb.org, an online target prediction database, we found that miR-214 can directly bind to the 3′UTR of CCNL2, which has previously been shown to be involved in the pathogenesis of PH and in the control of cell proliferation^15^. As shown in Fig. 1A, the seed sequences in the 3′UTR of CCNL2 and mature miR-214 were schematically compared, and the full-length 3′UTR of CCNL2 was amplified and cloned into the pcDNA3.0 vector downstream of a luciferase gene (labeled as pcDNA3-CCNL2-WT-3′UTR) (Fig. 1A). In addition, seed sequences within the predicted miR-214 target site were mutated in the construct (labeled as pcDNA3-CCNL2-Mut2-3′UTR) (Fig. 1A). Then, hPASMCs were co-transfected with the wild-type vector, the mutant vector, or the control luciferase vector together with the pRL-TK Renilla luciferase vector and miRNA mimics. We found that co-transfection of the miR-214 mimics suppressed the luciferase activity of the vector carrying wild-type CCNL2 3′UTR by approximately 50% (Fig. 1B). However, mutation of the predicted miR-214 target site in the 3′UTR of CCNL2 consistently abrogated the repressive effect of miR-214. These results indicated that miR-214 suppresses the translation of its target gene CCNL2 by binding to a seed sequence in the 3′UTR of CCNL2.

**Figure 3:**
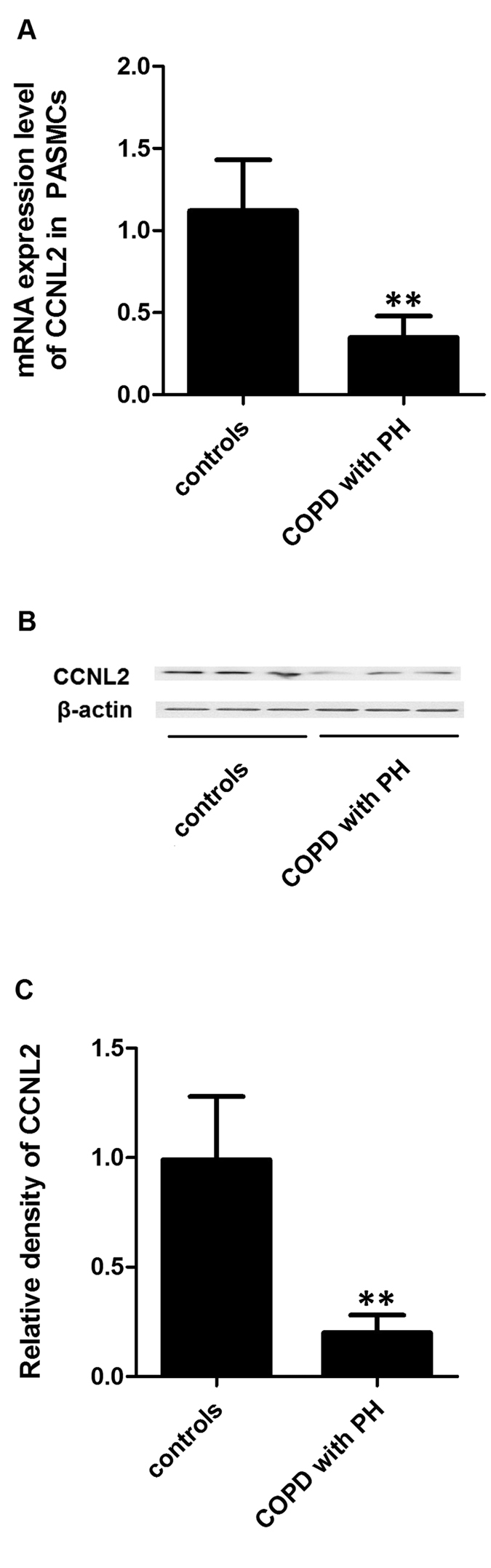
The expression pattern of CCNL2 was determined in PASMCs collected from COPD patients with or without PH and normal controls by using real-time PCR (**A**), western blot (**B,C**) densitometry analysis). (**P < 0.05 compared with the scramble controls).

**Figure 4:**
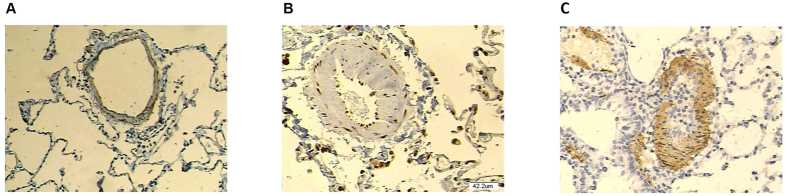
The protein expression level of CCNL2 was determined in lung tissue samples collected from COPD patients with PH (**A**), normal controls (**B**) and non-familial PH without COPD (**C**) by using immunohistochemistry.

We also examined the mRNA and protein expression levels of CCNL2 in hPASMCs from COPD patients with and without PH. Our results revealed decreased mRNA and protein expression of CCNL2 in hPASMCs from COPD patients with PH (Fig. 3A–C). These data suggest that miR-214 may be involved in the development of PH in COPD by targeting CCNL2. To confirm the above results, we used immunohistochemistry to determine the expression of CCNL2. We found that CCNL2 expression was significantly downregulated in remodeled arteries in COPD patients. In contrast, in remodeled arteries collected from the non-familial PH patients without COPD, the expression levels of CCNL2 were comparable to those in the controls (Fig. 4).

### Effects of hypoxia and miR-214 mimic/inhibitor treatment on the expression of CCNL2

**Figure 5:**
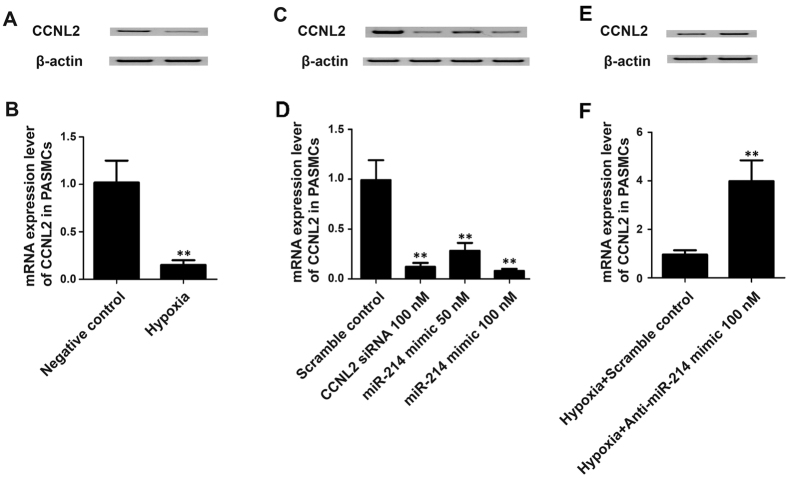
The expression levels of CCNL2 were determined in PASMCs exposed to normoxia and hypoxia by using western blot (**A**) and real-time PCR (**B**); The expression levels of CCNL2 were determined in PASMCs transfected with scramble controls, 100 nM anti-CCNL2 siRNA, 50 nM and 100 nM miR-214 mimics by using western blot (**C**) and real-time PCR (**D**); The expression levels of CCNL2 were determined in PASMCs exposed to hypoxia transfected with scramble controls or 100 nM anti-miR-214 inhibitors by using western blot (**E**) and real-time PCR (**F**). (**P < 0.05 compared with the scramble controls).

We subsequently examined the effect(s) of hypoxia and miR-214 mimic/inhibitor or anti-CCNL2 siRNA treatment on the mRNA and protein levels of CCNL2 in hPASMCs using real-time PCR and western blot, respectively. As shown in Fig. 5A,B, exposure to hypoxia significantly decreased the expression of CCNL2 in hPASMCs. Moreover, treatment with anti-CCNL2 siRNA or the miR-214 mimic (50 nM or 100 nM) significantly downregulated the mRNA and protein expression of CCNL2. However, treatment with anti-miR-214 inhibitors significantly restored the expression of CCNL2 in hPASMCs exposed to hypoxia (Fig. 5E,F).

### PASMC Proliferation was reduced upon transfection with anti-miR-214 inhibitors

**Figure 6:**
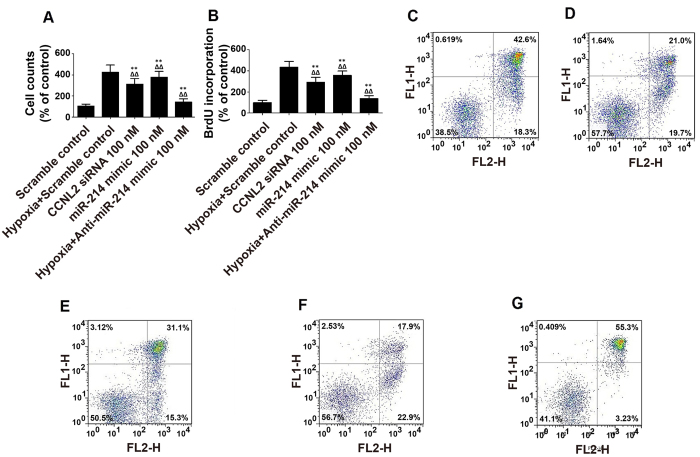
(**A**) The proliferation was determined in of PASMCs treated with scramble control, hypoxia and scramble, anti-CCNL2 siRNA, miR-214 mimics and hypoxia and anti-miR-214 inhibitors by cell counting; (**B**) The proliferation was determined in of PASMCs treated with scramble control, hypoxia and scramble, anti-CCNL2 siRNA, miR-214 mimics and hypoxia and anti-miR-214 inhibitors by BrdU cooperation assay; The apoptosis index was determined in PASMCs treated with scramble control (**C**), hypoxia and scramble (**D**) anti-CCNL2 siRNA (**E**) miR-214 mimics (**F**) hypoxia and anti-miR-214 inhibitors (**G**). (FL1 and FL2 are the gates set up to detect PI and annexin, respectively) (**P < 0.05 compared with the scramble controls; ΔΔP < 0.05 compared with hypoxia-exposed the scramble controls).

PASMC proliferation is an essential step of vascular remodeling in PH. Therefore, we addressed whether transfection with miR-214 mimics/inhibitors or with a target gene-specific siRNA affects PASMC proliferation as assessed by cell counting and BrdU assays. As shown in Fig. 6A,B, PASMC proliferation was significantly enhanced in cells exposed to hypoxia and was similarly promoted by transfection with anti-CCNL2 siRNA or miR-214 mimics. Conversely, application of anti-miR-214 inhibitors partially but significantly blocked the abnormal, hypoxia-induced enhancement of hPASMC proliferation.

**Figure 7:**
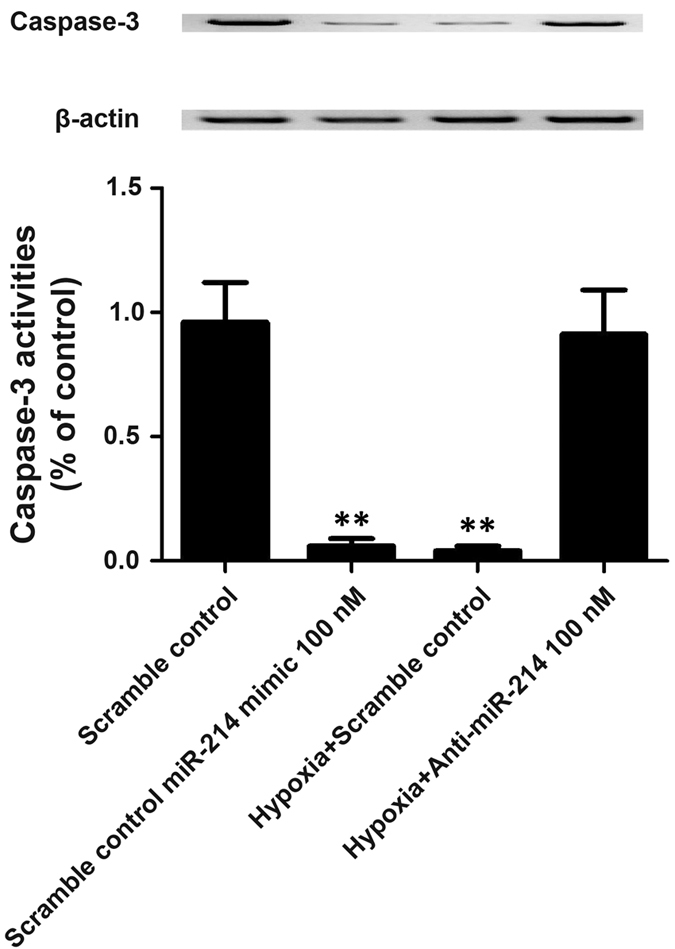
The protein expression level (**A**) and activity (**B**) of caspase 3 were determined in PASMCs treated with scramble control, miR-214 mimics, hypoxia plus scramble control, and hypoxia plus anti-miR-214 inhibitors. (**P < 0.05 compared with the scramble controls).

Furthermore, to explore the molecular mechanism underlying the modulation of PASMC proliferation, we performed flow cytometry analysis and found transfection with miR-214 mimics or anti-CCNL2 siRNA similarly inhibited apoptosis that was induced by treatment with H_2_O_2_. In contrast, transfection with anti-miR-214 inhibitors significantly suppressed PASMC proliferation by inducing apoptosis (Fig. 6C–G). Simultaneously, we measured the expression levels and activities of caspase-3 in PASMCs exposed to hypoxia or transfected with miR-214 mimics or inhibitors. We found that the caspase-3 protein and activity levels were consistently downregulated in the cells exposed to hypoxia or transfected with miR-214 mimics. However, this hypoxic effect was significantly attenuated by transfection with anti-miR-214 inhibitors (Fig. 7A,B).

### *In vivo* effect of anti-miR-214 treatment on the expression of CCNL2 and hypoxia-induced vascular remodeling

**Figure 8:**
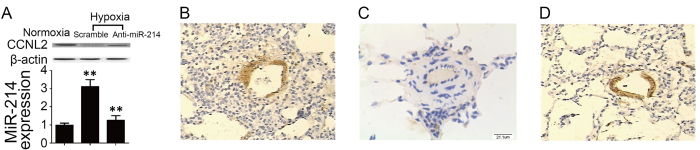
The *in vivo* effect of anti-miR-214 on the expression of CCNL2 and the development of hypoxic PH were examined in the animal model. (**A**) Hypoxia exposure substantially increased miR-214 expression and decreased CCNL2 expression accordingly (**A**), and treatment with anti-miR-214 restored the expression of CCNL2 and attenuated the hypoxia induce vascular remodeling (**B**) Mice exposed to normoxia; (**C**) Mice exposed to hypoxia; (**D**) Mice treated with anti-miR-214. (**P < 0.05 compared with the scramble controls).

To study the effect of anti-miR-214 on the expression of CCNL2 and the development of hypoxic PH, we established an animal model of PH by exposing mice to hypoxia and intratracheally administered a plasmid carrying anti-miR-214 or a control plasmid premixed with EXGEN500/5% glucose. PASMCs were isolated from the pulmonary vessels, and the expression marker GFP was equivalently detected in the plasmid-treated groups but was undetectable in the control group (data not shown). Furthermore, the expression levels of miR-214 and CCNL2 were determined in each group, and we found that exposure to hypoxia substantially increased miR-214 expression and decreased CCNL2 expression (Fig. 8A). However, treatment with anti-miR-214 restored the expression of CCNL2 and attenuated hypoxia-induced vascular remodeling (Fig. 8B–D).

## Discussion

COPD is a major cause of mortality and morbidity worldwide and is often complicated by the development of PH^16^,^17^,^18^. Although a wide variety of factors have been reported to be involved in the pathogenesis of PH, hypoxia is still believed to be the major cause of PH^19^. In the process of PH development, vascular remodeling, which is largely characterized by vascular smooth muscle cell proliferation and medial hypertrophy, contributes to a sustained elevation of pulmonary vascular resistance and pulmonary arterial pressure^20^,^21^. As major regulators of cellular activities, miRNAs have been implicated in the pathogenesis of hypoxia-induced PH^12^. In this study, we produced evidence that miR-214 is differentially expressed in PASMCs harvested from COPD patients with PH and that miR-214 can enhance the proliferation of PASMCs by targeting CCNL2.

It has previously been reported that miR-214 is significantly upregulated in the human cardiovascular system in response to hypoxia or ischemia^22^. In another study, hypoxia was found to regulate cellular behavior in colon cancer by modulating the ErbB2/ErbB3 signaling pathway synergistically with the upregulation of miR-214, although in an miRNA-independent manner. A recent study showed that miR‐214 protects the mouse heart from ischemic injury by controlling Ca^2+^ overload and cell death^23^. It has also been reported that the expression of miR-214 is sensitive to treatment with H_2_O_2_ and that miR-214 can protect cardiocytes by suppressing apoptosis mediated by H_2_O_2_^24^. miR‐214 expression in injured arteries in rats is highly upregulated, as confirmed by qRT‐PCR and Northern blot analyses^25^,^26^. Another study showed that the expression of miR‐214 is significantly upregulated in the infarct area and the border area of rat hearts 6 h after AMI^27^. These studies have demonstrated that the expression of miR‐214 confers resistance to cell apoptosis, and this evidence suggests that miR‐214 is an anti‐apoptotic factor. In this study, we confirmed that exposure to hypoxia significantly upregulated the expression of miR-214 in PASMCs and that the expression of miR-214 was substantially enhanced in PASMCs harvested from COPD patients with PH compared with those from the control group. Furthermore, using an online target prediction tool, we identified CCNL2 as a potential target of miR-214. Using a luciferase assay, we validated CCNL2 as an effective target of miR-214, and this result was further confirmed by the observation that transfection with miR-214 mimics significantly decreased the mRNA and protein expression levels of CCNL2 in PASMCs.

Cyclins are believed to play an important role in the regulation of cell proliferation. Certain cyclins have been identified as critical modulators of transcription^28^,^29^. CCNL2 differs from all other members of the cyclin family because it contains a C-terminal RS domain^30^,^31^; this domain is a hallmark of many proteins involved in the control of pre-mRNA processing, such as serine/arginine-rich splicing factors (SR proteins), which are characterized by the presence of at least one RNA-binding motif, and SR protein-related polypeptides^32^. CCNL2 has also been reported to be involved in the regulation of cell cycle progression and in the signal transduction of the apoptosis pathway^32^. Li *et al*. observed that ectopic overexpression of CCNL1 exerted direct anti-proliferative effects on BCG823 gastric cancer cells and enhanced the chemosensitivity of these cells to fluorouracil, docetaxel and cisplatin by inducing cell cycle arrest and cell apoptosis^33^. These observations were consistent with the results of cell cycle and apoptosis analyses of SMMC7721 human hepatocellular carcinoma cells^34^. In this study, we found that CCNL2 expression was significantly upregulated in response to hypoxia or the downregulation of miR-214, which suppresses the proliferation of PASMCs by inducing apoptosis. Consistently, downregulation of CCNL2 in PASMCs via transfection with miR-214 mimics significantly promoted cell proliferation by inhibiting apoptosis. Yang *et al*. observed that CCNL2 exerts direct antiproliferative effects on A549 human lung adenocarcinoma cells by inducing cell apoptosis *in vitro* and that overexpression of CCNL2 induced an increase in caspase-3 in A549 cells at 24 h post-transfection compared with mock vector transfection^35^. In this study, we found that upregulation of CCNL2 increased the caspase-3 protein expression and catalytic activity levels.

The observation that administration of anti-miR-214 inhibitors only partially ameliorated the hypoxia-induced proliferation of PASMCs indicates that other miRNAs that have never been documented in the literature or that do not necessarily perfectly match the 3′UTR of their target genes might be functionally involved in PASMC proliferation. Alternatively, other molecular mechanisms, such as methylation or polymorphisms, might be involved in the development of the disease.

Taken together, the results of our study showed for the first time that the abnormal, hypoxia-induced enhancement of PASMC proliferation can be partially but significantly attenuated by the downregulation of miR-214. This finding might shed some light on the molecular mechanism underlying the development of PH in COPD patients, as well as on the prevention and treatment of this complication.

## Additional Information

**How to cite this article**: Liu, H.T. *et al*. Upregulation of MicroRNA-214 Contributes to the Development of Vascular Remodeling in Hypoxia-induced Pulmonary Hypertension Via Targeting CCNL2. *Sci. Rep.* **6**, 24661; doi: 10.1038/srep24661 (2016).

## Figures and Tables

**Table 1:** Demographic and clinicopathological characteristics of the participants of this study.

	COPD + PH (N = 18)	Normal Control (N = 15)	P value
Age (years)	65.8 ± 6.8	64.3 ± 6.4	0.522
Gender (F/M)	3/15	4/11	0.484
Height (cm)	168.4 ± 9.3	169.2 ± 10.1	0.814
Weight (kg)	74.2 ± 13.2	76.6 ± 12.4	0.597
BMI (kg/m^2^)	26.2 ± 6.5	27.6 ± 7.3	0.564
FEV_1_ (%)	42.2 ± 8.9	99.4 ± 8.4	P < 0.001
FVC (%)	71.5 ± 14.5	98.5 ± 9.8	P < 0.001
TLC (%)	91.2 ± 16.3	118.2 ± 17.3	P < 0.001
DLCO (%)	62.3 ± 19.2	122.3 ± 23.5	P < 0.001
